# The IUPHAR/BPS Guide to PHARMACOLOGY in 2020: extending immunopharmacology content and introducing the IUPHAR/MMV Guide to MALARIA PHARMACOLOGY

**DOI:** 10.1093/nar/gkz951

**Published:** 2019-11-06

**Authors:** Jane F Armstrong, Elena Faccenda, Simon D Harding, Adam J Pawson, Christopher Southan, Joanna L Sharman, Brice Campo, David R Cavanagh, Stephen P H Alexander, Anthony P Davenport, Michael Spedding, Jamie A Davies, NC-IUPHAR

**Affiliations:** 1 Deanery of Biomedical Sciences, University of Edinburgh, Edinburgh EH8 9XD, UK; 2 Medicines for Malaria Venture, Post Box 1826, 1215 Geneva 15, Switzerland; 3 Institute of Immunology and Infection Research, University of Edinburgh, Edinburgh EH9 3FL, UK; 4 School of Life Sciences, University of Nottingham Medical School, Nottingham NG7 2UH, UK; 5 Experimental Medicine and Immunotherapeutics, University of Cambridge, Cambridge CB2 0QQ, UK; 6 Spedding Research Solutions SAS, Le Vésinet 78110, France

## Abstract

The IUPHAR/BPS Guide to PHARMACOLOGY (www.guidetopharmacology.org) is an open-access, expert-curated database of molecular interactions between ligands and their targets. We describe significant updates made over the seven releases during the last two years. The database is notably enhanced through the continued linking of relevant pharmacology with key immunological data types as part of the IUPHAR Guide to IMMUNOPHARMACOLOGY (www.guidetoimmunopharmacology.org) and by a major new extension, the IUPHAR/MMV Guide to Malaria PHARMACOLOGY (www.guidetomalariapharmacology.org). The latter has been constructed in partnership with the Medicines for Malaria Venture, an organization dedicated to identifying, developing and delivering new antimalarial therapies that are both effective and affordable. This is in response to the global challenge of over 200 million cases of malaria and 400 000 deaths worldwide, with the majority in the WHO Africa Region. It provides new pharmacological content, including molecular targets in the malaria parasite, interaction data for ligands with antimalarial activity, and establishes curation of data from screening assays, used routinely in antimalarial drug discovery, against the whole organism. A dedicated portal has been developed to provide quick and focused access to these new data.

## INTRODUCTION

**Figure 1.**
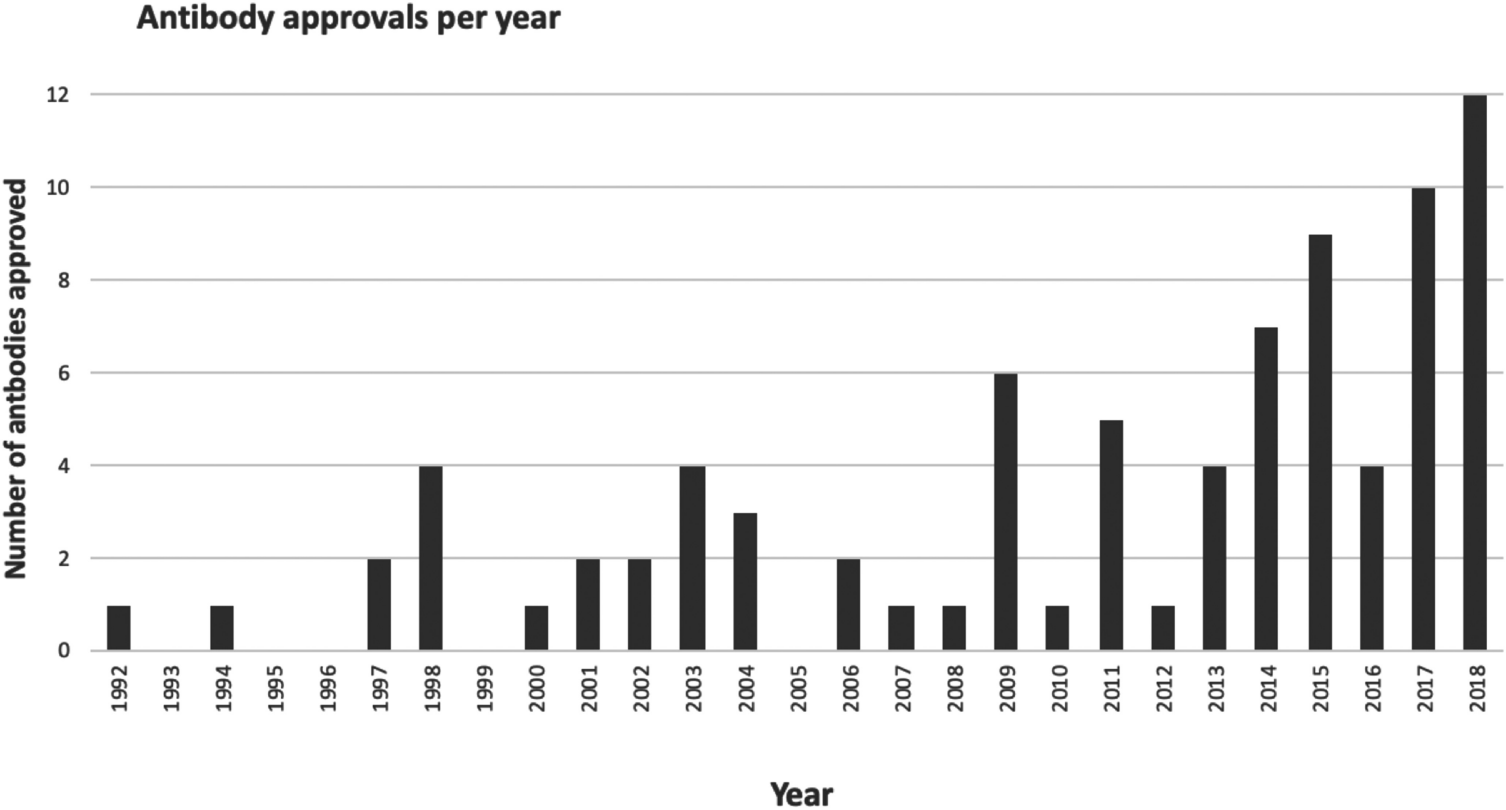
Growth in approvals of monoclonal antibodies over the last 26 years. Data include approvals for monoclonal antibodies and immunoglobulin Fc domain-containing fusion proteins that act at human protein targets and which are used to treat human diseases. Approval status is derived from the FDA (U.S. Food & Drug Administration; https://www.fda.gov), EMA (European Medicines Agency; https://www.ema.europa.eu) and EMC (Electronic Medicines Compendium; https://www.medicines.org.uk) websites, as well as from published ‘First global approval’ articles, and other online resources. The earliest approval date is used for each drug. The data set does not include the increasing number of biosimilar products that have been approved in the last 5–6 years.

The International Union of Basic and Clinical Pharmacology (IUPHAR) and the British Pharmacological Society (BPS) have jointly developed the Guide to PHARMACOLOGY (shortened to GtoPdb) from the antecedent IUPHAR-DB, a resource focused on receptors and channels that was first compiled in 2003 (1–3), and the BPS ‘Guide to Receptors and Channels’ (4), a compendium, providing concise overviews of the key properties of a wider range of targets than those covered in IUPHAR-DB. Incorporating these two resources, GtoPdb first came online in 2011, and expanded over the next 4 years, increasing its coverage of target families and quantitative target-ligand interactions. Expert guidance and oversight is maintained through the Nomenclature Committee of the International Union of Basic and Clinical Pharmacology (NC-IUPHAR) and its 109 subcommittees. From 2015, it extended into the priority area of immunopharmacology through the Wellcome-trust funded IUPHAR Guide to Immunopharmacology (GtoImmuPdb; www.guidetoimmunopharmacology.org) (5–7). The funding period for GtoImmuPdb concluded in October 2018, at which point the extension was officially launched at a focused immunopharmacology meeting, the report of which is available here (https://www.guidetoimmunopharmacology.org/pdfs/Edinburgh_Immunoparmacology_Meeting_Report_2018.pdf). GtoImmuPdb delivers a knowledge base that connects immunology with pharmacology (8), providing vital support to the research and development of drugs targeted at modulating immune, inflammatory or infectious components of disease. In the preceding ∼20 years, increasing numbers of antibodies and small molecule drugs that target the immune system have been approved as licensed medicines (Figure 1), and this corroborates the importance of immunopharmacology. The merit of extending GtoPdb into immune-relevant areas is strengthened by the partnership between IUPHAR and the International Union of Immunological Sciences (IUIS) to create standard tools and nomenclature.

Since our 2018 NAR publication (7), we have undertaken a further extension of the database; this time to include new types of data relevant to malaria pharmacology. Malaria is a major global health challenge with a disproportionate impact on resource-limited countries. In 2017, there were an estimated 219 million cases of the disease, leading to 435 000 deaths worldwide, with over 90% of these occurring in the World Health Organization's Africa Region (9). Action is taken against the spread and progress of the disease at many levels, from control of the mosquito vector to prevention and treatment with antimalarial drugs (10). Resistance to these drugs has emerged in many regions of the world, and strenuous efforts are being made to develop alternative drugs or vaccines. The Medicines for Malaria Venture (MMV; www.mmv.org) is an international product development partnership, dedicated to identifying, developing and delivering new antimalarial therapies that are both effective and affordable for the communities they are intended to protect (11). In the last twenty years, MMV and their partners have developed thirteen new medicines, some specialized for paediatric use or use in pregnant women, and have taken over stewardship of some drug development programmes from the Drugs for Neglected Diseases Initiative (www.dndi.org) (12–14). Recognizing the value of having a high-quality expert-curated database when designing drug development strategies, IUPHAR and MMV have collaborated to create the Guide to Malaria PHARMACOLOGY (abbreviated to GtoMPdb), a web resource specifically designed for malaria pharmacology.

This paper summarizes the main updates to the core GtoPdb and GtoImmuPdb, and then discusses in more detail the major new extension for malaria pharmacology.

## GUIDE TO PHARMACOLOGY AND IMMUNOPHARMACOLOGY CURATION AND EXPANSION

The GtoPdb remains unique in its approach to curation, which involves expert judgement at all stages. Our ∼100 NC-IUPHAR subcommittees (https://www.guidetopharmacology.org/GRAC/ContributorListForward#contributors), comprising ∼560 scientists worldwide, when combining their knowledge and expertise with that of our in-house curation team (www.guidetopharmacology.org/about.jsp#curation), providing a robust model of content selection and quality control.

### Curation and content

Since our last update in 2018 (7), the curation effort has focused on immunopharmacology. In addition to incorporating new immuno-relevant material, existing content has been enriched through annotating against immunological data types (described below). The selection of content for curation was supported through the identification of key articles and literature reviews by NC-IUPHAR subcommittees. The focus was on immunology and inflammation literature that identified lead compounds, their molecular targets and contained evidence of pharmacological modulation and relevance to human disease. Other useful resources include pharmaceutical companies’ declared development programmes, key papers circulated on Twitter and analysis of patents to identify pharmacological data beyond peer-reviewed publications. We also review clinical trial registries and applications to the WHO for new INNs (International Nonproprietary Name) (which provides an indication of developments in the immunity/inflammation/immuno-oncology fields), and monitor new drug approvals.

#### Targets

**Figure 2.**
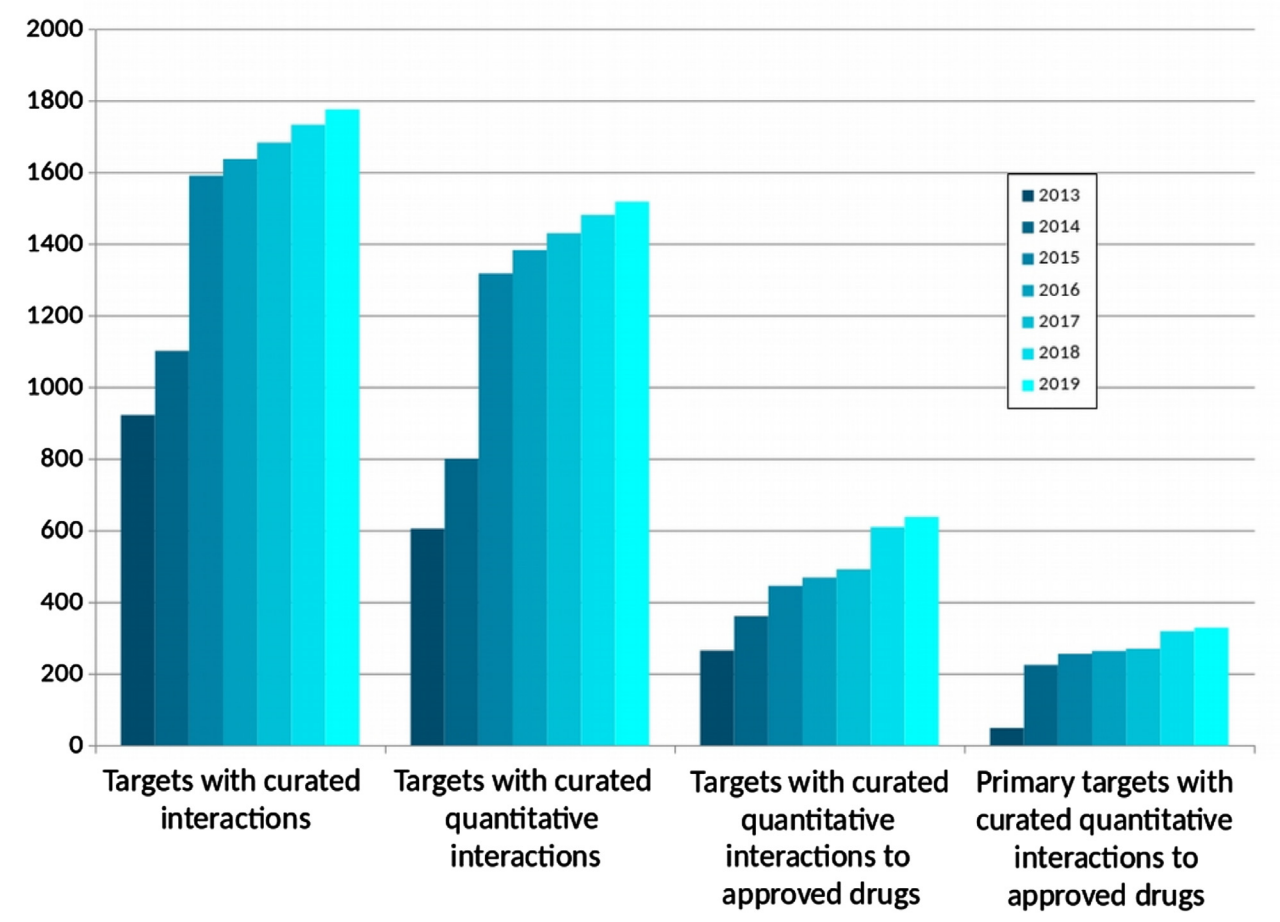
Growth of human target interaction data in GtoPdb since 2013.

The expansion in GtoPdb content is summarised in Table 1. Individual targets in GtoPdb use a UniProtKB/SwissProt (15) Accession as their primary identifier and are organised into hierarchical target families. These are organised into top-level target classes (Table 1A). GtoPdb contains 1781 human protein targets with curated ligand interactions (Table 1C), 1525 of these targets have quantitative binding data. If the count of interactions is restricted to just those human targets with quantitative binding data to an approved drug, these number 638 interactions, 329 of which are cases where the protein is designated as the primary target of that drug (Figure 2).

**Table 1.** Guide to Pharmacology data counts for targets, ligands and interactions from database release 2019.4

	GtoPdb 2020 (± 2018)	GtoImmuPdb 2020 (± 2018)
**A. Target class content. Human UniProtKB accession Counts**		
GPCRs	398 (+3)	100 (+14)
Nuclear Hormone Receptors	48 (0)	8 (+2)
Catalytic Receptors	249 (+6)	114 (+7)
Ion Channels	278 (+4)	40 (+20)
Transporters	539 (+30)	9 (+4)
Enzymes	1212 (+28)	196 (+62)
Other proteins	207 (+33)	103 (+39)
Total number of targets	2932 (+98)	568 (+148)**
**B. Ligand category counts**		
Synthetic organics	6524 (+717)	754 (+305)
Metabolites	585 (+1)	38 (+15)
Endogenous peptides	790 (+8)	197 (+21)
Other peptides including synthetic peptides	1338 (+41)	52 (+15)
Natural products	266 (+19)	15 (+6)
Antibodies	261 (+28)	156 (+35)
Inorganics	39 (+1)	1 (0)
Approved drugs	1442 (+108)	264 (+56)
Withdrawn drugs	71 (+4)	14 (+3)
WHO essential list	193 (+193)	36 (+36)
Ligands with INNs	2353 (+239)	476 (+140)
PubChem CIDs*	7319 (+617)	758 (+274)
PubChem SIDs*	9714 (+736)	1183 (+367)
Total number of ligands	9803 (+825)	1213 (+397)
**C. Interaction counts**.		
Human targets with ligand interactions	1781 (+97)	528 (+138)
Human targets with quantitative ligand interactions	1525 (+166)	452 (+131)
Human targets with approved drug interactions	638 (+75)	212 (+60)
Primary targets*** with approved drug interactions	329 (+16)	116 (+25)
Ligands with target interactions	8464 (+801)	1081 (+363)
Ligands with quantitative interactions	7455 (+739)	880 (+327)
(approved drugs)	925 (+101)	189 (+51)
Ligands with clinical use summaries	2436 (+347)	597 (+174)
(approved drugs)	1439 (+107)	263 (+55)
Number of binding constants	48531 (+2043)	-
References	36440 (+4707)	-

*Counts taken prior to submitting to PubChem post-2019.4 release.

**604 targets are tagged in GtoImmuPdb, but 36 have no Human UniProtID.

***Primary target indicates the dominant Molecular Mechanism of Action (MMOA).

The table includes a comparison to the figure in the 2018 update (7). Categories are not mutually exclusive, and targets and ligands can fall into more than one category.

The curation effort for immunopharmacology has seen updates to established targets such as histamine receptors (https://www.guidetoimmunopharmacology.org/GRAC/FamilyDisplayForward?familyId=33) and anti-histamine drugs, glucocorticoid receptors (https://www.guidetoimmunopharmacology.org/GRAC/ObjectDisplayForward?objectId=625&familyId=98&familyType=NHR) and anti-inflammatory glucocorticosteroid drugs, cyclooxygenase enzymes (https://www.guidetoimmunopharmacology.org/GRAC/FamilyDisplayForward?familyId=269) and NSAID drugs, and pattern recognition, cytokine and chemokine receptor families. Families of pattern recognition receptors that were previously absent from the GtoPdb have been added (16). Given the pharmaceutical industry's interest in harnessing the power of the immune system against cancer (immuno-oncology), curation effort was applied to include the novel areas of endogenous immune checkpoint proteins and their ligands, and to the addition of therapeutic immune checkpoint modulators. This allowed the generation of a new ligand family ‘Immune checkpoint modulators’ (https://www.guidetopharmacology.org/GRAC/FamilyDisplayForward?familyId=969) that provides users with a single, easy access point to the GtoPdb list of those drugs that are already in use and many more currently under clinical or preclinical investigation.

#### Immunological data types

As previously described (7), GtoImmuPdb was designed to incorporate immunological data types and associate these with existing targets and ligands. This enrichment includes immunological processes, cell types and diseases. The methodology for process and cell type inclusion was described in our last update but, in summary, we have used Gene Ontology (17,18) biological process terms and Cell Ontology (19) cell types to underpin top-level categories against which we have annotated protein targets (Table 2). The use of ontologies gives a controlled vocabulary and external identifiers; these support interoperability. In the case of processes, they also prove a route to auto-curate some associations via UniProt-GO (15) associations. Users are able to view detailed lists of targets associated with the top-level categories, and linked ontology terms are listed alongside curator comments on an individual target's relevance to immunopharmacology.

**Table 2.** GtoImmuPdb Process and Cell Type categories showing the number of human protein targets in the database annotated with them

Process	Annotated human targets
Barrier integrity	49
Inflammation	633
Antigen presentation	142
T cell (activation)	196
B cell (activation)	161
Immune regulation	503
Tissue repair	19
Immune system development	251
Cytokine production & signalling	504
Chemotaxis & migration	256
Cellular signalling	476
Cell type	Annotated human targets
B cells	51
Dendritic cells	41
Granulocytes	46
Innate lymphoid cells	6
Macrophages	56
Mast cells	39
Natural killer cells	26
Other T cells	3
Stromal cells	1
T cells	76

#### Ligands

Our criteria for including ligands continues to prioritise those that are well characterised (e.g. with triangulation from multiple sources) and that possess quantitative interactions with protein targets (Table 1B). In the last two years, we have added over 800 new ligands, with around 48% being relevant to immunopharmacology (reflecting the curation focus in this area). We have added a new ligand category that includes those in the WHO essential medicine list, currently 193 ligands, 36 of which are included in GtoImmuPdb.

To capture novel drug mechanisms that are being pursued by the pharmaceutical industry, we have selected exemplars that highlight novel pharmacological approaches that expand upon existing drug classes. A number of PROTAC (proteolysis targeting chimera) type drugs are in development. Rather than simply acting as inhibitors, PROTACS direct their protein target to proteasomal degradation. As examples we have included JH-XI-10-02 (a CDK8 kinase-directed degrader; www.guidetopharmacology.org/GRAC/LigandDisplayForward?ligandId=9887) and ARD-69 (an androgen receptor degrader; www.guidetopharmacology.org/GRAC/LigandDisplayForward?ligandId=10182), both of which have postulated anti-tumour potential (20,21).

New classes of anti-inflammatory and analgesic drugs are also being developed, with examples such as naproxcinod (a NO-donating naproxen analogue prodrug; www.guidetoimmunopharmacology.org/GRAC/LigandDisplayForward?ligandId=9551) and ATB-346 (a hydrogen sulfide-releasing naproxen derivative; www.guidetoimmunopharmacology.org/GRAC/LigandDisplayForward?ligandId=9534), which have both progressed to clinical evaluation as novel therapeutics for arthritis and osteoarthritis, respectively (22,23).

#### PubChem submissions

**Figure 3.**
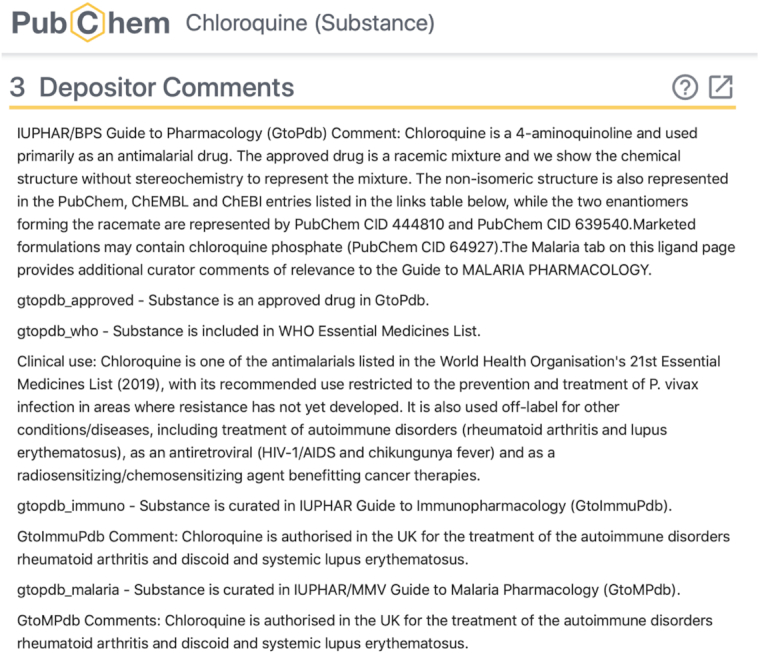
Ligand chloroquine, depositor comments from PubChem substance record (https://pubchem.ncbi.nlm.nih.gov/substance/178102177#section=Depositor-Comments). Displaying depositor comments added during submission from GtoPdb. These indicate inclusion in either GtoImmuPdb or GtoMPdb; drug approval status; and curatorial comments.

The range of powerful utilities arising from GtoPdb ligand data integration into PubChem (24) and the rest of the NCBI system (25), including PubMed and PDB ligands, has already been described (7). Users are able to make domain-specific queries related to both immunopharmacology and malaria pharmacology via curatorial tagging within the depositor comment sections in the substance records (SIDs) (Figure 3). This allows queries to be executed from both the PubChem Substance (SID) and PubChem Compound (CID) interfaces (26), by using an advanced search of ‘comments’ fields, as outlined in Table 3. PubChem Substances are community-provided compounds, and many entries may exist for the same molecule. Each may contain different information about the molecule, depending on the information provided by the submitter. PubChem extracts the unique chemical structures from Substance records (standardization) and stores them as PubChem Compounds. This means that substance records from different data sources about the same molecule are aggregated in a common Compound record in PubChem. The wide range of GtoPdb-associated data mining that can be executed in PubChem is too large to be expanded in detail but selected worked examples have been described (27). Informative statistics represented in the 7612 total CIDs (2019.4 release) include the following:

- 66% conform to Lipinski's Rule-of-Five (28,29) lead-like property limits.
- 148 entries are unique to GtoPdb as their only annotation source (as of 1 October 2019).
- 78% have an exact match to an automated patent extraction source (not all of these are currently claimed structures but many can be linked to SAR data sets in patent documents to which links will be added).
- 726 do not have BioAssay Tested results. This means GtoPdb is the only source of literature-extracted activity for those compounds.
- 19% have a PDB ligand match.
- 27% have been annotated with the MeSH terms included in the ‘Pharmacologic Actions’ classification (https://www.ncbi.nlm.nih.gov/mesh/68020228). By definition we have thus greatly expanded the annotation of these compounds with pharmacological data.
- 77% have a vendor match.
- 25% do not have a match to PubChem ChEMBL entries.

**Table 3.** Counts of GtoPdb tagged ligands in PubChem (1 October 2019; data from GtoPdb 2019.4 release)

Query	PubChem Substance query	SID counts
All ligands in GtoPdb	‘IUPHAR/BPS Guide to PHARMACOLOGY’[SourceName]	9806
Approved drugs	AND gtopdb_approved	1337
Clinical antibodies	AND antibody	258
GtoImmuPdb	AND gtopdb_immuno	964
GtoMPdb	AND gtopdb_malaria	72
Query	PubChem Compound query	CID counts
All ligands in GtoPdb	‘IUPHAR/BPS Guide to PHARMACOLOGY’[SourceName]	7612
Approved drugs	SID > CID	1234
GtoImmuPdb	SID > CID	798
GtoMPdb	SID > CID	72

Apart from the total selected by source, all other CID numbers are retrieved via the ‘Find related data’ > Select PubChem Same Compound (i.e. a SID > CID conversion).

**Figure 4.**
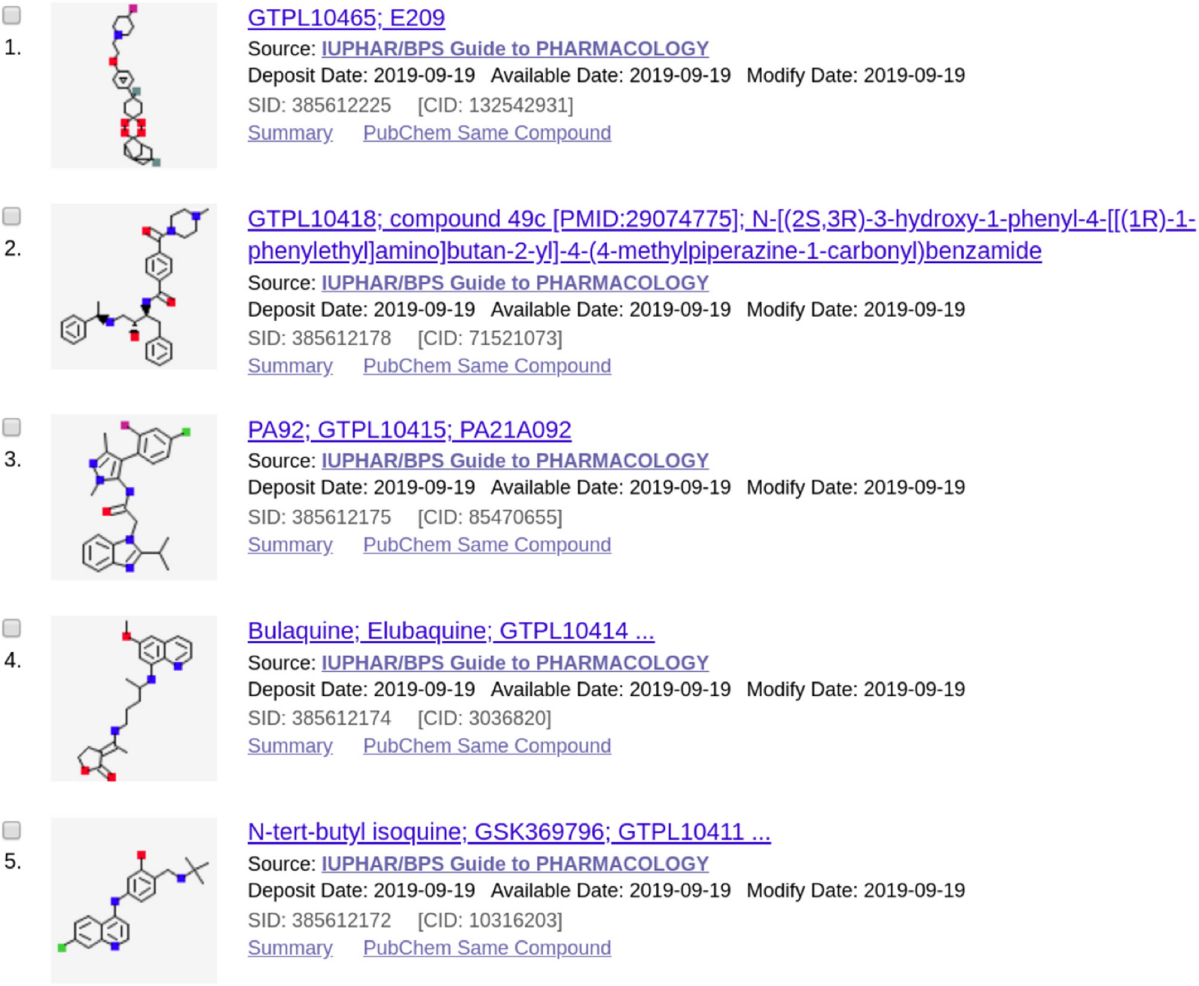
Top results returned when searching PubChem Substance for GtoPdb compounds included in GtoMPdb ((‘IUPHAR/BPS Guide to PHARMACOLOGY’[SourceName]) AND gtopdb_malaria; https://www.ncbi.nlm.nih.gov/pcsubstance/?term=(%22IUPHAR%2FBPS+Guide+to+PHARMACOLOGY%22%5BSourceName%5D)+AND+gtopdb_malaria).

As mentioned a new ‘Guide to Malaria Pharmacology’ tag has been added to the depositor comments in our PubChem submission. This means that the set of antimalarial compounds in GtoPdb (2019.4 release) can be retrieved from PubChem (Figure 4), and specific statistics for 72 CIDs can be generated:

- 44 conform to Lipinski's Rule-of-Five (28,29) lead-like property limits.
- 57 have an exact match to an automated patent extraction source.
- 19 do not have BioAssay Tested results.
- 15 have a PDB ligand match.
- Only 27 have been annotated with the ‘Antimalarials’ MeSH term.
- 56 have a vendor match.
- 16 do not have a match in ChEMBL entries.

### Website

#### Disease summary pages

**Figure 5.**
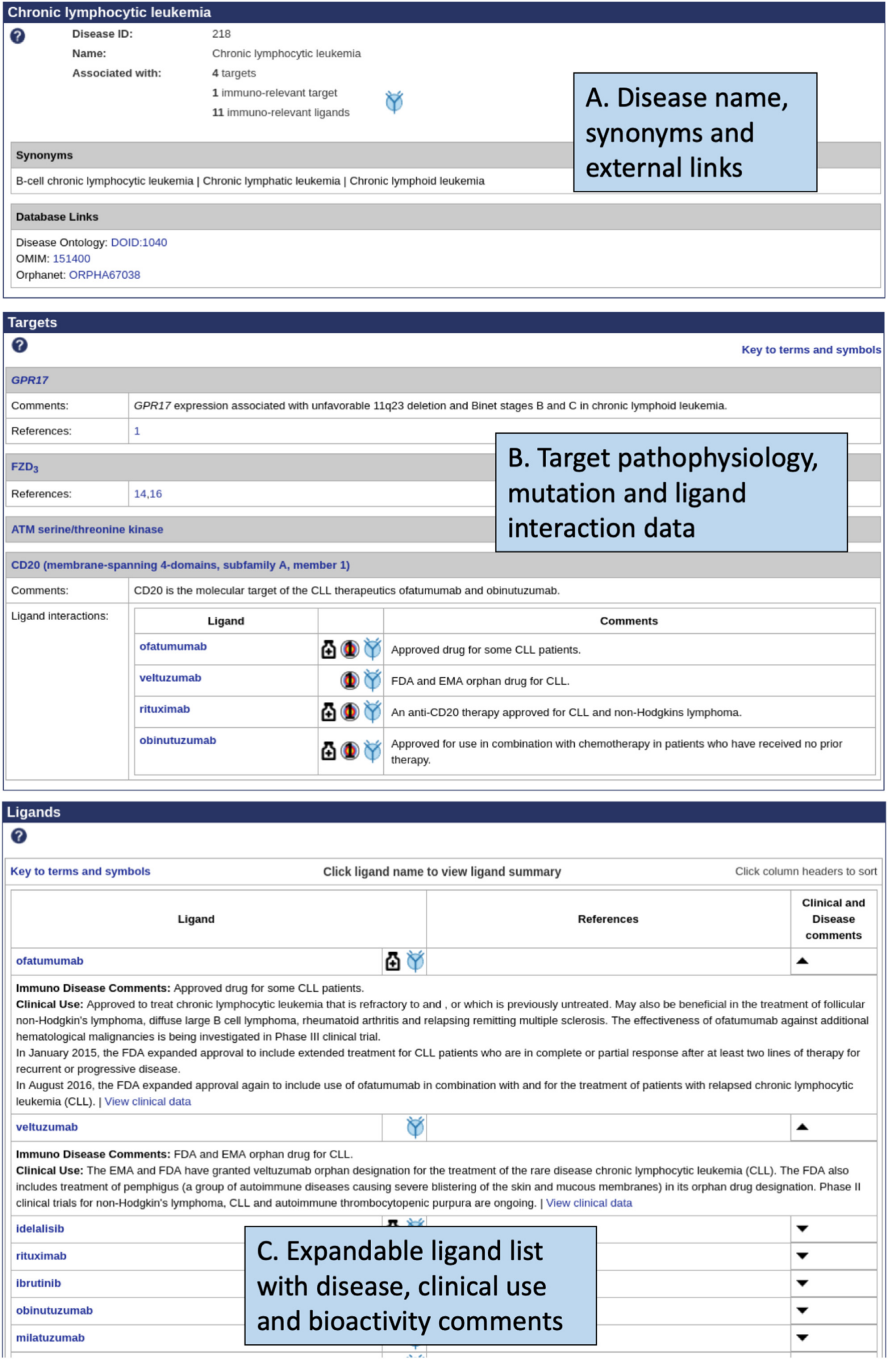
GtoPdb disease summary page for chronic lymphocytic leukemia (https://www.guidetoimmunopharmacology.org/GRAC/DiseaseDisplayForward?diseaseId=218). (**A**) Summary of disease and counts of associated targets and ligands. (**B**) Associated target data section. (**C**) Associated ligand data section, with expandable drop-down comments.

The website has been significantly enhanced by the development of disease summary pages (Figure 5). As part of the GtoImmuPdb extension, targets have been linked to ligands of immunological relevance to the diseases in which they play a role. The GtoPdb already contained limited disease information, which included pathophysiologies and clinically-relevant mutations for targets, but it was not organised in a manner to find all relevant data for each disease easily. The new development has allowed the consolidation of all the associated data and to present this to users on a single disease page. In total, there are over 1000 disease entries in the database linked to targets and/or ligands. For all diseases, there are external links to one or more entries in OMIM (30,31), Orphanet (https://www.orpha.net) and the Disease Ontology (32) and list any known synonyms, which helps users to cross-check between different resources.

The target section of the disease summary page displays information on targets associated with the disease. Ligands that are associated with the disease, that also have interaction data with the target, are listed alongside curatorial comments and indications if they are approved drugs. If there is further data on the targets role and any other therapeutically-used drugs acting via the target, these are also displayed.

The ligand section lists all the drugs and other compounds associated with the disease, the majority of which come from the curation effort for GtoImmuPdb. Users can expand this section for additional information linking the ligand to the disease, such as clinical use and bioactivity comments.

#### WHO essential medicines

GtoPdb now includes an additional category of ligands: those currently included in the World Health Organization (WHO) Model List of Essential Medicines (21st, 2019; https://www.who.int/medicines/publications/essentialmedicines/en/). These ligands are displayed under the ‘WHO’ category on our ligand list page (https://www.guidetopharmacology.org/GRAC/LigandListForward?type=WHO-essential&database=all) and on individual ligand summary pages, inclusion in this list is marked under the classification section.

#### Move to hypertext transfer protocol secure (HTTPS)

In order to improve and protect the integrity of GtoPdb, we have completed the transition from HTTP to HTTPS. This helps to make end user communication more secure.

#### Chemicalize Pro (ChemAxon)

A key feature of the website is the ability to perform searches by chemical structure (http://www.guidetopharmacology.org/GRAC/chemSearch.jsp). The chemical structure search tool utilises Marvin JS by ChemAxon. In the 2019.1 (January 2019) release, the API control was updated to use Chemicalize Pro (https://pro.chemicalize.com/). This update simplifies the integration of Marvin JS into our website.

#### Page navigation

Many of the pages on GtoPdb, particularly the detailed target view pages, are rich in information, requiring users to scroll far down the page, away from the main menu bar that is used for navigation. We have therefore implemented a new drop-down navigation bar, which is revealed when users scroll-down on longer pages. This keeps key menu items, and most importantly the site search, in focus at all times.

## GUIDE TO MALARIA PHARMACOLOGY

The Guide to Malaria Pharmacology (GtoMPdb) project was initiated in October 2017, with funding from MMV, to expand the content of the existing database to include antimalarial ligands and targets. This required extension of the GtoPdb to display the new data and to augment existing search and browse functions. A purpose-built portal has been developed, in consultation with key opinion leaders in malaria research, to provide optimized access to the antimalarial data. This initiative has enriched the GtoPdb and will hopefully advance innovation by providing, in a single expert-curated database, results from antimalarial drug discovery programmes and the scientific literature. The full public release of the GtoMPdb was made in September 2019.

### Curation and content

The focus for the GtoPdb has hitherto been the pharmacology and immunopharmacology associated with human non-infectious diseases. Targets identified and validated in *Plasmodium*, the genus of protozoan parasite known to cause malaria, had not previously been included in the GtoPdb. Indeed, there were very few, if any, data from non-mammalian species in the GtoPdb. Similarly, the number of ligands with antimalarial activity was small. In extending the parent database to capture data of relevance to malaria pharmacology our curatorial procedures have been reviewed, allowing the identification of modifications required to the database schema and web interface. The sections below will expand on these aspects but an important consideration has been to utilize GtoPdb entity IDs rather than generating a separate identifier system. This is because malaria pharmacology is a subcategory of pharmacology, which is in accordance with the approach taken with the previous GtoImmuPdb expansion. Instead, a project specific tag has been introduced to identify ligands and targets of interest to malaria pharmacology and that are to be included in the GtoMPdb.

The established curation protocol employed for the GtoPdb (described in previous updates and reviewed in (33)) required modest adaptations to allow capture of antimalarial ligands and targets as database entries. Papers were selected for curation when they had: an explicit chemical structure and at least one of the following; quantitative potency for antimalarial activity *in vitro*, activity data (where available) against a purified *Plasmodium* target, and *in vivo* and/or clinical data. This content was added with guidance from a group of scientific advisors and an expert advisory committee (www.guidetomalariapharmacology.org/malaria/gtmpAbout.jsp#contributors): expanding to 30 *Plasmodium* molecular targets and over 70 ligands annotated with antimalarial activity, including approved drugs, clinical candidates and research leads. In addition, antimalarial medicines are presented that were used in the past but are now either no longer recommended or are restricted in their use. These medicines have often become less effective over time because of the emergence and geographical spread of strains of the parasite that have become resistant to them. A summary of GtoMPdb data content is provided in Table 4, and full antimalarial target (www.guidetomalariapharmacology.org/GRAC/FamilyDisplayForward?familyId=970) and ligand (www.guidetomalariapharmacology.org/GRAC/LigandListForward?type=AntiMal&database=all) lists are accessible on the website.

**Table 4.** GtoMPdb data counts for targets, ligands and interactions (database release 2019.4)

Guide to Malaria Pharmacology Content Breakdown	
Targets	30
Ligands	72
Ligands (approved drugs)	19
Ligands (WHO essential lists)	17
Targets with quantitative ligand interactions	20
Targets with approved drug interactions	4
Ligands with interactions	67
Ligands with interaction to known targets	38
Ligands with interaction to unknown targets	34
Ligands with quantitative interactions (approved drugs)	65 (18)
Ligands with clinical use summaries (approved drugs)	39 (19)

#### Targets

Antimalarial targets is a new target family, encompassing targets identified and validated in the *Plasmodium* parasite. It has been introduced as a subfamily under other protein targets, within the hierarchical classification of target families used by GtoPdb. In accordance with GtoPdb targets, individual *Plasmodium* targets use a UniProtKB/SwissProt Accession as their primary identifier. The nomenclature adopted is derived from information available from PlasmoDB (www.plasmodb.org), a genome database for the genus *Plasmodium* that is part of the EuPathDB Bioinformatics Resource Center (www.eupathdb.org) (34,35). The pragmatic decision was taken to link the gene symbol to the *P. falciparum* 3D7 reference sequence (36) at PlasmoDB, although information for other *Plasmodium* species and other *P. falciparum* clones is also curated. Similarly, UniProt IDs are assigned to antimalarial targets using the *P. falciparum* 3D7 reference proteome (www.uniprot.org/proteomes/UP000001450) (15).

#### Ligands

All GtoMPdb tagged ligands are now included in the antimalarial ligands family, a new group of related ligands that has been added to the GtoPdb ligand family list. In line with GtoPdb curation protocol, most chemical structures have been resolved to PubChem entries and all the curated antimalarial compounds have been submitted to PubChem where they acquire GtoPdb-specific Substance Identifiers (SIDs). The criteria for inclusion of a ligand has however been adapted because many antimalarial ligands do not possess quantitative interactions with a known protein target.

#### Comparison with ChEMBL content

A major utility offered by GtoMPdb is the prompt surfacing of new lead compounds, their metadata and associated molecular targets from the literature, but its value is also strengthened by the relative lack of similar open-access resources that curate data on malaria pharmacology. Indeed, comparing our coverage with that of ChEMBL (37), highlights a few distinctions. For our 30 *Plasmodium* targets, only six have UniProt IDs in common with ChEMBL. We suggest two reasons for this, the first being we have picked up more recent publications. The second is the challenge to assign a UniProt ID based only on the author's target description in their paper. This is compounded by the problem that even the reference strain *P. falciparum* 3D7 has only 165 proteins annotated to the Swiss-Prot standards whereby naming equivocality is reduced (but cannot be eliminated) but for all *P. falciparum* isolates there are over 125 000 sequence entries. We thus speculate that the targets-in-common between GtoMPdb and ChEMBL might be higher than the distinct UniProt IDs indicate. For ligands, the difference is smaller in that 20% of GtoMPdb structures have no match in ChEMBL. In some cases, ChEMBL may have chosen a different isomer, depending on their interpretation of the image in the paper, but we can also once again attribute some of this difference to the more recent publications we have extracted. As an example, PubMed ID:30894487 (38), was published in April 2019 and the lead (ligand ID 10398; www.guidetomalariapharmacology.org/GRAC/LigandDisplayForward?ligandId=10398) entered PubChem via our June 2019 release.

### Modifying the GtoPdb schema for malaria pharmacology

**Figure 6.**
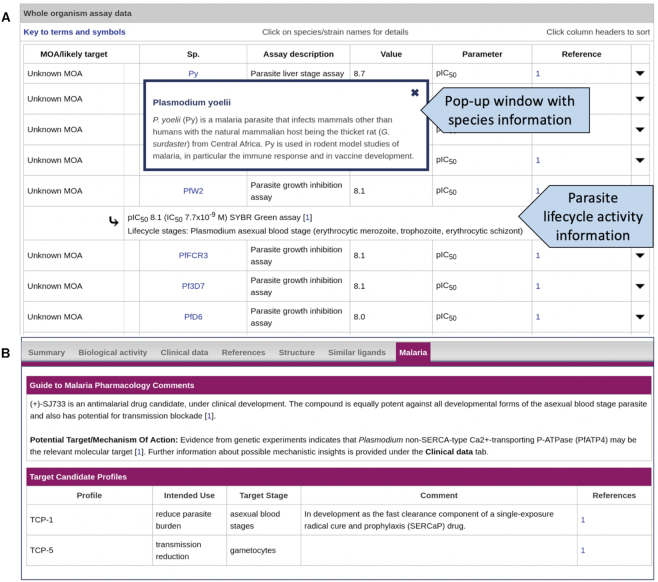
GtoMPdb ligand summary page illustrating new interface features. (**A**) The modified interactions table provides data from whole cell assays, with information available for the parasite lifecycle stage activity (given in the dropdown information for each row) and *Plasmodium* species (pop-up window in the species column). The table shown is for a ligand with an unknown target (ganaplacide, GtoPdb Ligand ID 9946; https://www.guidetomalariapharmacology.org/GRAC/LigandDisplayForward?tab=biology&ligandId=9946). (**B**) The malaria tab displays curator comments of particular relevance to malaria, including summary information about the potential target or mechanism of action, and a table containing TCP details (example shown is (+)-SJ733, GtoPdb Ligand ID 9723; https://www.guidetomalariapharmacology.org/GRAC/LigandDisplayForward?tab=malaria&ligandId=9723#malaria).

A number of changes have been made to the database structure and web interface to improve both the capture and presentation of antimalarial data. To fully describe the activity and target interactions of antimalarial compounds requires the display of additional information, some of which is specific for the malaria parasite. We have extended the database schema and modified the interactions table display to accommodate these requirements (see Figure 6A). A new ‘whole organism’ assay type has been introduced to capture data from the high-throughput whole cell screens used routinely in antimalarial drug discovery (39). As already mentioned, many antimalarial compounds have a poorly understood mechanism of action and an unknown molecular target.

The *Plasmodium* parasite has a complex lifecycle (40), and the goal to eradicate malaria will require the discovery and development of new antimalarial medicines that target multiple stages of the parasite lifecycle and in particular the transmission cycle (39,41,42). Capturing quantitative interaction data for the activity of an antimalarial ligand against the different developmental forms of the parasite was identified as a key requirement for the GtoMPdb. With guidance from the group of scientific advisors, a set of top-level lifecycle stages was defined (see www.guidetomalariapharmacology.org/GRAC/ParasiteLifecycleStagesForward for further details) against which ligand interactions can now be annotated in the database. These are collective categories, for one or more developmental forms of the parasite, and which form the basis of organising, navigating and searching for parasitic lifecycle activity data. The interactions table display has been modified to include this information (see Figure 6A).

### Accessing and viewing antimalarial data

#### Portal

A new dedicated portal has been developed, giving open and optimized access to the antimalarial data in the GtoPdb. In designing and building this portal, we have drawn on our experience from the GtoImmuPdb project, with the provision of a dedicated domain for the GtoMPdb (www.guidetomalariapharmacology.org) and the design of a distinct identity (logo, menu-bar and colour scheme). In addition to the existing ligand and target browse/search functionality available on the parent GtoPdb site, customized views of the data have been developed that include parasite lifecycle and target species activity. These have been designed in consultation with malaria researchers to deliver data returns that they find the most useful. Access to all data is from the menu-bar or from panels on the portal homepage. The site search, available in the top-right corner of all database pages, has been extended to incorporate the new antimalarial data. The search algorithm has also been modified for queries from GtoMPdb pages to increase the ranking of results for entities with the malaria project tag. A description of navigating and viewing data using the GtoMPdb portal is provided in the following subsections.

#### Targets

The targets panel links directly to the new antimalarial targets family. From there, the user can browse and navigate to individual target pages and find detailed information about each target and its interactions. For individual target pages, the overall style and layout used by the parent database was retained with the intention of providing a familiar interface for users who are already acquainted with the GtoPdb. A new comments section has been added, allowing information of relevance to malaria to be highlighted.

#### Ligands

The ligands panel provides customized access to the ligand lists page, with tabs for different categories of antimalarial ligands in the database, such as approved antimalarial drugs and antimalarial ligands with a Target Candidate Profile (TCP). The TCP describes the key attributes of a new molecule from the MMV global malaria portfolio, as it enters clinical development (41,42).

For individual ligand summary pages, we have again retained the style and layout of the parent database. A new malaria tab has been implemented (Figure 6B) and this is displayed on the summary page for GtoMPdb tagged ligands. This view provides information of relevance to malaria pharmacology and a separate table containing TCP information (where this is available). A link to the GtoPdb ligand family page, with the antimalarial ligands category highlighted (www.guidetomalariapharmacology.org/GRAC/LigandFamiliesForward), is available from the menu-bar.

#### Parasite lifecycle stages

**Figure 7.**
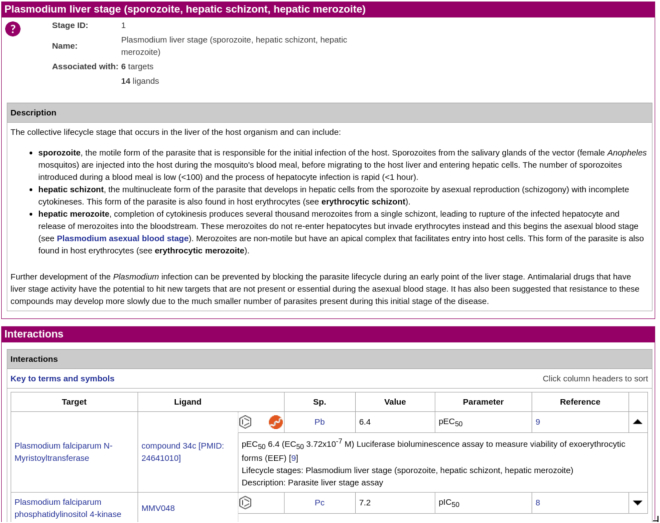
Example of a parasite lifecycle page, here the *Plasmodium* liver stage (https://www.guidetomalariapharmacology.org/GRAC/ParasiteLifecycleDisplayForward?stageId=1), developed specifically for GtoMPdb.

The parasite lifecycle stages panel is one of the tailored routes developed for accessing antimalarial information in the GtoMPdb. Clicking on the home page link takes the user to a summary page that includes general information about the lifecycle of the *Plasmodium* parasite and a list of our top-level lifecycle stage categories. The same list of links to top-level categories is provided in the parasite lifecycle stages panel and from the menu-bar. Clicking on one of these categories links to a new page providing a description of the lifecycle stage and all interactions contained in the database that are associated with it (Figure 7).

#### Target species

**Figure 8.**
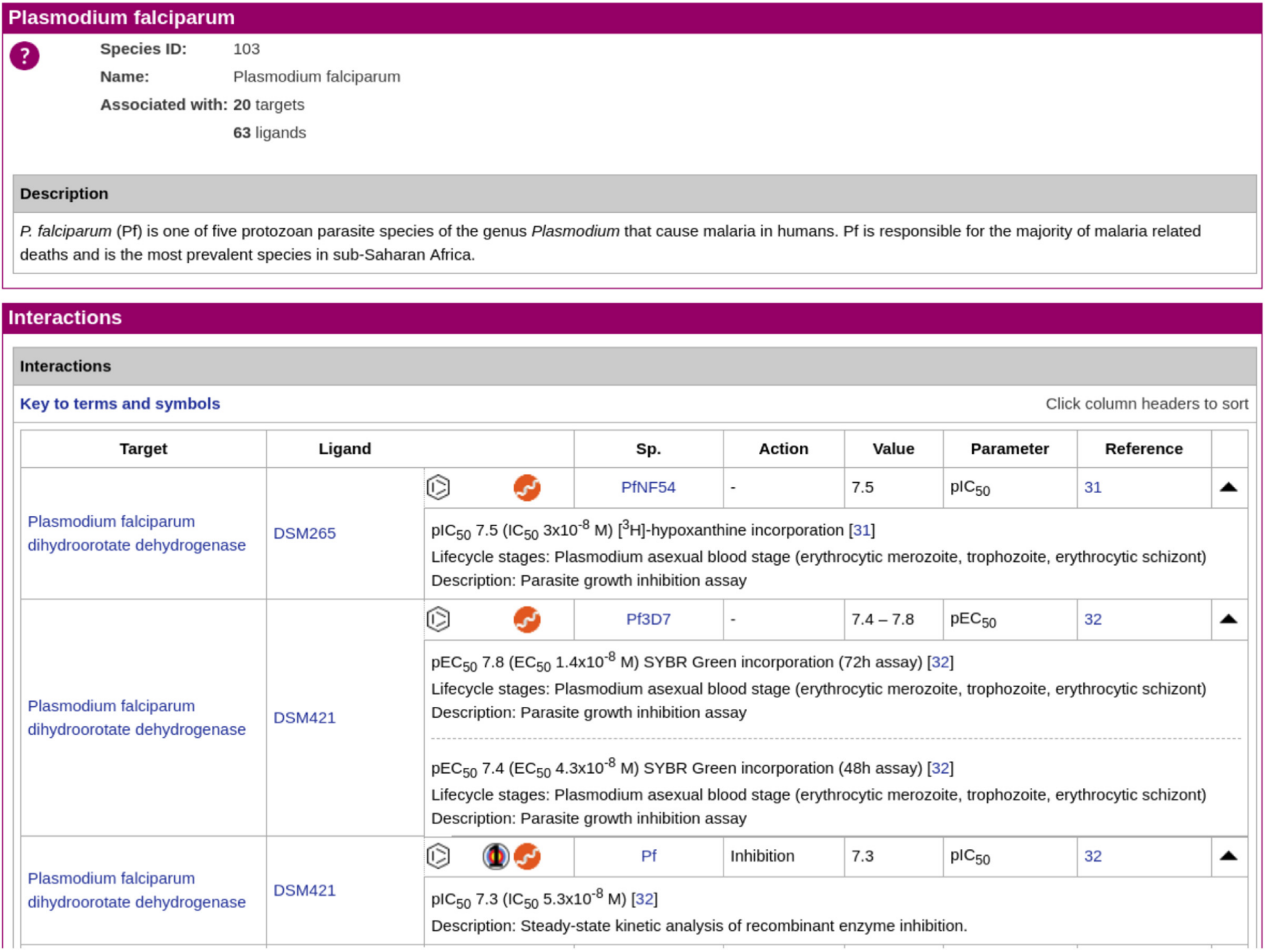
Example of an individual target species page, here showing *Plasmodium falciparum* (https://www.guidetomalariapharmacology.org/GRAC/MalariaTargetSpeciesDisplayForward?speciesId=103), developed specifically for GtoMPdb.

The target species panel is another new route for accessing antimalarial information. Clicking on the target species home page link takes the user to a summary page that includes background information about *Plasmodium* species that are of clinical or experimental importance, links to individual pages for *Plasmodium* species for which data are presented in the GtoMPdb, and a list of useful resources. The same list of species links is provided in the target species panel and from the menu-bar. Clicking on one of these species links takes the user to a new page providing a description of the species and all interactions contained in the database that are associated with it (Figure 8).

#### Resources

The portal homepage provides links to a number of supplementary resources, including a news panel and links to the GtoPdb Twitter feed. Help pages developed specifically for the GtoMPdb (www.guidetomalariapharmacology.org/malaria/gtmpHelpPage.jsp) can be accessed from the menu-bar. In addition, popup windows provide tailored help for each of the data panels on the portal homepage and have been made available on other pages for data fields that require further explanation.

## COLLABORATIONS, CONNECTIVITY AND INTEROPERABILITY

### Elixir-UK

GtoPdb remains one of the ELIXIR-UK node services, under its human health and disease strategic theme (https://elixiruknode.org/human-health-and-disease/). Our involvement with ELIXIR-UK brings closer ties with other key UK bioinformatics resources and facilitates collaboration on the use of standard ontologies and identifiers. This is valuable as we continue seeking to ensure GtoPdb is a FAIR-compliant (Findable, Accessible, Interoperable, Reusable) resource.

### Immunopaedia

We have recently begun to build links with Immunopaedia (www.immunopaedia.org.za), an open-access online platform for immunology education (43). The resource aims to improve engagement between core immunology and clinical practice, and it is the official International Union of Immunological Societies (IUIS) learning site. Immunopaedia provides clinical case studies to help highlight immunological concepts, online courses to support teaching and learning in immunology, and also provides information on treatment and diagnostics on infectious diseases. We have added links from ligand summary pages to relevant case studies at Immunopaedia. Immunopaedia has reciprocally implemented links from a number of case studies to the corresponding GtoPdb target and ligand pages.

### SynPharm

Links are now in place on appropriate target and ligand pages to SynPharm (http://synpharm.guidetopharmacology.org/), a database of drug-responsive protein sequences (44), derived from the interactions in GtoPdb and using data from the RCSB Protein Data Bank (PDB) (45). SynPharm is an open-access, web-based tool that integrates pharmacological and ligand–protein binding information to present data on the drug-binding domains of proteins in a manner useful to synthetic biologists. Its strengths as a bioinformatic tool to facilitate the design of drug-responsive proteins are demonstrated in a recent paper that discusses engineering druggability into the Cpf1 effector of CRISPR gene editing (46).

### Tocris

As a trusted supplier of life science reagents, we have established a sponsorship collaboration with Tocris Bioscience (https://www.tocris.com/), to add links to their catalogue from GtoPdb ligand summary pages. Currently there are 1357 ligands with links to the Tocris website records for these compounds.

### Database links and interoperability

We maintain a list of database link-outs from GtoPdb target and ligand pages and this can be viewed at www.guidetopharmacology.org/helpPage.jsp#databaseLinks. Since 2018, we have added links to Immunopaedia and PlasmoDB, and updated our out-links to ChEMBL (37), thereby adding ∼800 new links.

The GtoPdb has recently been included in the External Links service at Europe PMC (EPMC) (https://europepmc.org/LabsLink). On EPMC pages, links to target and ligand entries have been added to the papers curated by GtoPdb that include a quantitative description of the ligand–target interaction. It is possible to retrieve all these references at EMPC by running an ‘Advanced Search’ and selecting ‘IUPHAR/BPS Guide to Pharmacology’ from the ‘External Links’ drop-down list (LABS_PUBS:‘1969’) as the cross-reference query. It is possible for this set of publications to be further combined, as Boolean-type queries, with all other types of EPMC indexing including Bibliographic Fields, Filters, Data Links, External Links and Annotations.

### External profile

Disseminating information about GtoPdb (as one of our routes to impact) is done in a number of ways. We regularly publish peer-reviewed articles covering database updates, curation challenges and guides to accessing data (https://www.guidetopharmacology.org/nciupharPublications.jsp#Database). We also share posters and presentations made by the team at national and international conferences. These are collated on our SlideShare account (www.slideshare.net/GuidetoPHARM). We regularly use Social Media (Twitter (@GuidetoPHARM), Facebook and LinkedIn) to announce database updates, publications of interest and alert followers to upcoming events. Our blog is used to provide detailed descriptions of database releases, technical descriptions of developments and commentaries on hot topics in pharmacology (https://blog.guidetopharmacology.org/category/hot-topics/). We track user interactions via Google Analytics, which shows a monthly average of ∼33 600 sessions from ∼22 000 users. The Concise Guide to PHARMACOLOGY 2017/18 (47) is the third edition in a series of biennial publications containing concise overviews of nearly 1800 human drug targets - the fourth edition is due for publication in late 2019.

## FUTURE DIRECTIONS

From its origins in 2011, the GtoPdb has become recognized as an authoritative, open resource that provides expert-curated molecular interactions between ligands and targets encoded by the human genome. As described in this update, the unique model of content selection and quality control provided by the GtoPdb has recently been extended to capture data for the priority areas of immunopharmacology and malaria pharmacology. In the immediate future, work will continue on increasing the content of both the GtoImmuPdb and GtoMPdb. For the GtoMPdb, new data curation will continue until June 2020, with a focus on expanding the antimalarial targets family. We will also address the need to provide a hierarchical organization of subcategories for this expanded family.

The initial development phase for the GtoMPdb database and web-application is now complete, but it will continue to be supported and maintained through core GtoPdb development resources. We will be seeking feedback from users and we aim to implement the most useful suggested improvements to the interface. In addition, work is planned to include all data for the GtoMPdb in our web-services output, and to provide advanced search tools specific to the malaria project.

For the core database, we are also working with Bioschemas (http://bioschemas.org/) (48) to add schema.org semantic mark-up to GtoPdb, which would make it simpler for search engines to index the website, and make it easier to collate and analyse the data. Our current focus is on implementing mark-up on all ligand summary pages, including properties from the Bioschemas MolcularEntity profile (https://bioschemas.org/specifications/drafts/MolecularEntity).

In the longer term, we are exploring other key areas of pharmacological interest with the intention of providing further expansion of the GtoPdb.

## DATA ACCESS

GtoPdb, GtoImmuPdb and GtoMPdb are available online at www.guidetopharmacology.org, www.guidetoimmunopharmacology.org and www.guidetomalariapharmacology.org, respectively. All three resources are licensed under the Open Data Commons Open Database License (ODbL) (www.opendatacommons.org/licenses/odbl/) and the contents are licensed under the Creative Commons Attribution ShareAlike 4.0 International (CC BY-SA 4.0, https://creativecommons.org/licenses/by-sa/4.0/). Advice on linking to us and for accessing and downloading data are provided here: https://www.guidetopharmacology.org/linking.jsp. GtoPdb aims to make three to four public database releases per year; the data summaries and statistics reported in this paper are from release 2019.4 (September 2019). Our downloads page (available from www.guidetopharmacology.org/downloads.jsp) provides a dump file of the full PostgreSQL database (http://www.postgresql.org/), in addition to a number of specific download files for targets, ligands, interactions, peptides, and (new in 2019) endogenous/natural ligands. We also provide RDF flat files, which users can load into a local triple store and perform SPARQL queries across the data. Our REST web services are available at http://www.guidetopharmacology.org/webServices.jsp and provide computational access to data in JavaScript Object Notation (JSON) format. We continue to respond to users’ requests and have extended the web-services to include transduction mechanisms, selectivity data and adding Ensembl Gene IDs into the interaction download. We encourage users to communicate with us if they download data in any format, both for further advice and so we are aware of applications using GtoPdb data.

## CITING THE RESOURCE

Please cite this article rather than the previous ones; citation advice for specific target pages appears on the website. Please refer to our resources on first mention by full correct name (IUPHAR/BPS Guide to PHARMACOLOGY, IUPHAR Guide to IMMUNOPHARMACOLOGY, IUPHAR/MMV Guide to MALARIA PHARMACOLOGY) including the capitalization. For subsequent abbreviation, please use GtoPdb, GtoImmuPdb and GtoMPdb, specifying the release version number (this can be found on our About page (http://www.guidetopharmacology.org/about.jsp#content)).

## Notes

Present address: Joanna L. Sharman, Novo Nordisk Research Centre Oxford, Novo Nordisk Ltd., Innovation Building - Old Road Campus, Roosevelt Drive, Oxford OX3 7FZ, UK.

Present address: Christopher Southan, TW2Informatics Ltd, Göteborg 42166, Sweden.
